# Supervised and non-supervised Nordic walking in the treatment of chronic low back pain: a single blind randomized clinical trial

**DOI:** 10.1186/1471-2474-11-30

**Published:** 2010-02-10

**Authors:** Jan Hartvigsen, Lars Morsø, Tom Bendix, Claus Manniche

**Affiliations:** 1 Clinical Locomotion Science, Institute of Sports Science and Clinical Biomechanics, University of Southern Denmark, Campusvej 55, DK-5230 odense M, Denmark; 2 Nordic Institute of Chiropractic and Clinical Biomechanics, part of Clinical Locomotion Science, Forskerparken 10A, DK-5230 Odense M, Denmark; 3 The Back Research Center, part of Clinical Locomotion Science, University of Southern Denmark, Lindevej 5, DK-5750 Ringe, Denmark

## Abstract

**Background:**

Active approaches including both specific and unspecific exercise are probably the most widely recommended treatment for patients with chronic low back pain but it is not known exactly which types of exercise provide the most benefit. Nordic Walking - power walking using ski poles - is a popular and fast growing type of exercise in Northern Europe that has been shown to improve cardiovascular metabolism. Until now, no studies have been performed to investigate whether Nordic Walking has beneficial effects in relation to back pain.

**Methods:**

A total of 151 patients with low back and/or leg pain of greater than eight weeks duration were recruited from a hospital based outpatient back pain clinic. Patients continuing to have pain greater than three on the 11-point numeric rating scale after a multidisciplinary intervention were included. Fifteen patients were unable to complete the baseline evaluation and 136 patients were randomized to receive A) Nordic walking supervised by a specially trained instructor twice a week for eight weeks B) One-hour instruction in Nordic walking by a specially trained instructor followed by advice to perform Nordic walking at home as much as they liked for eight weeks or C) Individual oral information consisting of advice to remain active and about maintaining the daily function level that they had achieved during their stay at the backcenter. Primary outcome measures were pain and disability using the Low Back Pain Rating Scale, and functional limitation further assessed using the Patient Specific Function Scale. Furthermore, information on time off work, use of medication, and concurrent treatment for their low back pain was collected. Objective measurements of physical activity levels for the supervised and unsupervised Nordic walking groups were performed using accelerometers. Data were analyzed on an intention-to-treat basis.

**Results:**

No mean differences were found between the three groups in relation to any of the outcomes at baseline. For pain, disability, and patient specific function the supervised Nordic walking group generally faired best however no statistically significant differences were found. Regarding the secondary outcome measures, patients in the supervised group tended to use less pain medication, to seek less concurrent care for their back pain, at the eight-week follow-up. There was no difference between physical activity levels for the supervised and unsupervised Nordic walking groups. No negative side effects were reported.

**Conclusion:**

We did not find statistically significant differences between eight weeks of supervised or unsupervised Nordic walking and advice to remain active in a group of chronic low back pain patients. Nevertheless, the greatest average improvement tended to favor the supervised Nordic walking group and - taking into account other health related benefits of Nordic walking - this form of exercise may potentially be of benefit to selected groups of chronic back pain patients.

**Trial registration:**

http://www.ClinicalTrials.gov # NCT00209820

## Background

Nordic Walking (NW) is fitness walking using specially designed poles for the purpose of activating the upper body during walking. The poles are equipped with rubber or spike tips and the walking itself resembles Nordic style skiing. Thus NW is a low tech and cheap form of exercise that can be done by practically anybody and therefore has an increasing number of users particularly in northern Europe [1]. By using the poles the muscles in the upper body can be activated and the length of each step taken is supposedly increased resulting in a faster gait [1]. Some controversy exist regarding the physiological effects of NW. According to earlier studies, NW appears to increase gait speed and cardiovascular metabolism [2,3] but results of a recent study showed that persons using NW poles walked at a slower pace when compared to persons walking without the poles, however persons performing NW had higher heart rate and higher oxygen consumption [4].

NW is also being used as a rehabilitation modality in various conditions such as intermittent claudication [5], Parkinson's Disease [6,7], depression [8], and after atheletic injuries [9] where general physical activity has been shown to be of benefit. It is still unclear to what extent NW improves cardiovascular fitness and whether there is an advantage over normal brisk walking [2,3,10] and a possible advantage is likely dependent on the intensity of the NW and the skill of the individual [4].

Low back pain (LBP) is a modern epidemic all over the western world and one of the leading causes of sick leave and disability [11]. In Denmark alone roughly half of the population has experienced LBP during the past year and of these, 20% had pain for more than 30 days during this period [12]. Such longer-lasting pain is a strong predictor of future pain and disability [13] which translates into enormous costs to society [11]. Naturally, strategies aiming at effectively preventing or treating LBP are urgently needed [14].

Among the more promising intervention strategies in relation to LBP is general physical activity. Physical activity is associated with a long range of health benefits [15] and it appears that an active lifestyle to some extent protects against LBP both in childhood [16], during working years [17] and in seniors [18]. Also in relation to treatment of persons already suffering from LBP, general physical activity may be of benefit [19], in fact general aerobic exercise may be as effective as many back specific exercise systems used either alone or in combination with other conservative treatments [17,20].

A particular challenge in interventions involving patient participation such as exercise therapy is the issue of compliance to the prescribed exercises [21]. Hayden et al concluded that training and exercise therapy was slightly more effective if delivered in groups and under supervision of an instructor [22]. One reason for the apparent advantage could be that non-supervised patients do not comply with instructions and thus do not get the physiological effects and ultimately do not improve.

The aim of the current study was to investigate these issues in a single blind randomized design by comparing the effect of supervised NW versus non-supervised NW versus advice to stay active in relation to pain and pain-related function in patients with chronic LBP referred to a specialized out-patient back pain clinic [23]. We hypothesized that supervised NW would improve pain and pain related function in chronic LBP patients when compared to unsupervised NW and advice to remain active. Furthermore we hypothesized that supervised NW would result in a greater average physical activity level when compared to unsupervised NW.

## Methods

### Participants

Patients were recruited from a secondary sector specialized out-patient back pain clinic and was conducted over a two-year period from August 2005 to August 2007. This clinic receives referrals from primary care physicians or primary care chiropractors when at least four weeks of treatment in primary care by a family physician, chiropractor, physical therapist, or a combination thereof has not resulted in satisfactory improvement. At the back clinic all patients received extensive examination and diagnostic procedures and information about self-care for back pain and attended group exercises twice a week for four weeks before being offered inclusion into this trial. To be included in the project participants

• had LBP with or without leg pain > 8 weeks

• had averaged pain > 3 during the past two weeks on the 11 point numeric rating scale

• had completed four weeks of treatment in the primary sector by a family physician, chiropractor, physical therapist, or a combination thereof

• had concluded all examinations, individual and group treatment at the back clinic with at least a 75% attendance rate

• were able to read and understand Danish

### Exclusion criteria were

• co-morbidity preventing patient from participating in the full intervention

• unable to sit on a stationary bike for at least 30 minutes in order to perform watt max bicycle test

For eligible participants interested in participating in this trial, baseline data were collected one week after ending the four week group exercise program.

### Randomization and interventions

Randomization was carried out by a project secretary after collection of the baseline data. Participants drew a sealed opaque envelope containing information about treatment allocation. Envelopes were arranged in clusters of 15 to secure an even spread over the three groups and was therefore an ongoing process over the two years of recruitment.

**Group A **received instruction and performed NW in groups of 6-8 twice a week for 8 weeks under supervision of a specially trained NW instructor. As soon as a group was filled the NW commenced resulting in different groups performing this intervention at different time points over the two years. There were three different scenic routes between three and four kilometres long. In order to determine the desired walking intensity in the supervised NW group, we placed accelerometers on the NW instructors for the first couple of sessions at the very beginning of the trial. By using, comparing, and averaging all the values from the instructors while they were performing NW with the first participants, an intensity interval arose that was used as reference. Each session lasted around 45 minutes and while encouraged to walk at the predetermined intensity, not all participants were able to comply. Thus, the participants had to be allowed to walk at different speeds and faster participants could to walk ahead and upon completion of the route, turn around, meet, and "pick up" the slower group in order to complete the session with them. Consequently the dose and frequency was equal for all participants but the intensity varied somewhat.

**Group B **was instructed in NW once by the same specially trained instructors in a single one-hour session. Afterwards they were left to perform NW as much as they wanted to at home on their own for the next 8 weeks.

**Group C **was given information about active living and exercise and about maintaining the daily function level they have achieved during the four week period at the back pain clinic by remaining active.

NW poles were provided free of charge to everyone included in the project. Participants randomized to group C received their poles as a gift after the 8 week intervention period but received no instruction in NW.

### Outcome measures

Primary outcome measures were

**Low Back Pain Rating Scale **(LBPRS) assesses the dimensions of pain, disability, and physical impairment for patients with LBP [24]. The pain index measures uses three 11-box numeric rating scales (pain now, worst and average pain during the last two weeks) for back and leg pain separately. Each response score is added giving a scale range of 0-60 points. The disability index comprises 15 items and possible answers to each question were "yes", "can be a problem", or "no" which were then scored as 0, 1, and 2 giving a range of 0-30 points

**Patient Specific Function Scale (PSFS) **assesses functional limitations in a variety of clinical presentations. Patients are asked to identify three important activities which they are having difficulty or are unable to perform because of their problem. In addition to specifying the activities, patients are asked to rate on an 11-box numeric rating scale the current level of difficulty associated with each activity [25]

Information on primary outcome measures were collected 11, 26, and 52 weeks after randomization.

Secondary outcome measures were

**EQ-5D **is a standardized 5-item generic measure of health related quality of life. Domains of mobility, self-care, usual activities, pain/discomfort, and anxiety are assessed using a three point response scale [26].

**Medication use, other treatment for LBP, time off **work was collected using questionnaires at different time points.

**Expectation to treatment **was collected, and all participants rated their expectations to each of the three groups on a five-point Likert scale with response options ranging from 1 *(very good) *to 5 *(poor)*.

### Accelerometer measurements

Participants in the supervised NW and unsupervised NW groups wore Actigraph GT 256 accelerometers (MTI) the fourth and fifth week of the eight week intervention period in order to gauge if there were differences in their physical activity levels. Participants were sent an MTI by mail and were asked to wear it every day for two weeks, and then return it in a pre-paid return envelope. A MTI is strapped around the waist near by the pelvis, as tight and as close to the skin as possible. It measures physical activity by registering the vertical movement of the point of gravity thereby both registering physical activity and, using the amplitude, also the intensity. The MTI registers physical activity in counts pr. minute which makes it possible to monitor activity precisely during the day and MTIs have been shown to reliably measure daily physical activity in different populations [27,28]. When analyzing data from MTIs, it is not possible to see exactly what kind of activities the user has been performing and only activities involving vertical motion are registered, i.e. the MTI does not register for example bicycling and swimming.

### Statistical analyses

Based on the primary outcome measures, a sample size of 130 participants would provide 80% power to detect a difference of eight units on the LBPRS (SD = 13) between the primary and each of the comparator groups, assuming alpha of 0.05 using a two-sided test. This change has been shown to be the minimal clinically important difference (MCID) for patients undergoing the standard treatment at the backcenter and data from this previous study was used as basis for the power calculations [29]. To allow for a 20% drop out the sample size was increased to 150.

For primary and secondary endpoint the focus was on two pair-wise comparisons between supervised and unsupervised NW and between supervised NW and the advice to remain active intervention. This was accomplished through an ANCOVA analysis with the change from baseline as dependent variable, the three treatment groups as a catagorical variable, and adjusting for baseline level as a continuous variable. Standard errors were estimated via the sandwich approach which is a useful method for obtaining robust standard errors in complex models. The pair-wise comparison was performed through a Wald test. The within group treatment effect was evaluated using a paired T-test.

Explorative analysis of other variables (being on sick leave, medication use, and receiving concurrent treatment) followed the same approach. Explorative analysis of dichotomous variables was performed in logisitic regression. Finally, in the exploratory analysis the primary end point was reevaluated. We defined a successful outcome if the change was equal to or greater than the MCID [29] and counted the number of participants in each group who achieved the MCID for the LBPRS pain and disability dimensions. The analysis of the influence of expectations on the outcome was performed in a regression model adjusting for individual baseline level. All analyses were performed using the Stata version 10 statistical software [30] and based on the intention to treat analysis set.

Data from the accelerometers were extracted and downloaded on computer. These files were analyzed using excel computer software and only data from participants contributing data for seven days or more were used. Between groups comparisons were described as mean and SD of the total average activity intensity per hour, comparison of high and low scores between groups and mean time spent at each intensity level during the day. Then activity level in the two groups were compared with the intent of showing possible differences in the level of physical activity in the group which performed supervised NW and the group performing NW on their own at home.

Statistical significance was accepted at p < 0.05. P-values > 0.05 but < 0.1 are referred to as borderline significant.

### Approval

The study was approved by the regional ethics committee for Funen and Vejle Counties, approval # VF 2005005

## Results

### Participants

**Figure 1:**
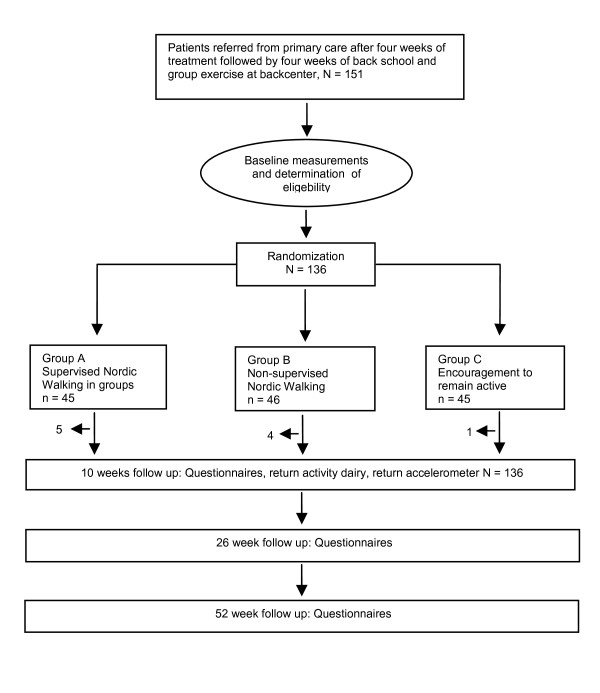
**Flow chart of study**.

Altogether 151 chronic LBP patients fulfilling the inclusion criteria were initially recruited. Fifteen patients were unable to comply with the baseline testing and dropped out prior to randomization and 136 patients were randomized. During the eight intervention weeks, five, four, and one participants dropped out of the groups and did not contribute with follow-up data at any point. The reasons for dropping out were primarily inability to comply with the intervention schedule. Thus 126 patients completed the intervention and contributed data to the analyses (Figure 1). Characteristics of included patients are summarized in Table 1. The randomization resulted in three groups comparable in all baseline variables including expectations to treatment (Table 1).

**Table 1:** Baseline charateristics of participants.

	Supervised NW	Unsupervised NW	Advice
**n**	45	46	45
**Age (years)**	49.2 (11.1)	45.4 (10.8)	45.5 (11.0)
**% female**	77.5	69.1	68.2
**Current smoker %**	28.9	30.9	40.9
Low Back Pain Rating Scale, pain*	46.1 (16.6)	50.7 (21.8)	47.3 (18.2)
Low Back Pain rating Scale, function*	44.4 (18.1)	47.3 (15.2)	48.9 (17.6)
Patient Specific Function Scale*	18.4 (6.1)	20.1 (4.2)	17.3 (5.4)
EQ5D*	67.5 (16.5)	62.7 (16.1)	63.9 (16.8)
On sick leave %**	47.4	60.0	63.6
**Pain medication almost daily %**	56.4	70.0	69.8
**Expectation of treatment**			
**To which randomized**	1.2 (0.5)	2.12 (0.8)	2.21 (0.9)
**- Supervised NW**	1.2 (0.5)	1.3 (0.5)	1.5 (0.7)
**- Unsupervised NW**	1.9 (0.7)	2.1 (0.8)	2.1 (0.7)
**- Advice to remain active**	2.26 (0.8)	2.54 (0.8)	2.21 (0.9)

*0-100 scale

** 2/1/0 participants excluded due to no labor market affiliation

### Primary outcome measures

**Figure 2:**
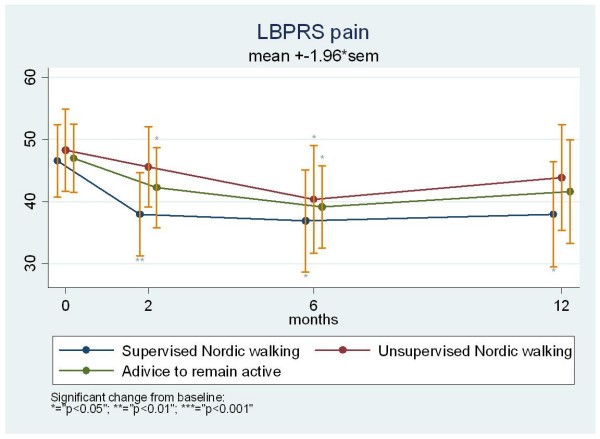
**Change over 12 months on the Low Back Pain Rating Scale (LBPRS) pain subscale for chronic low back pain patients randomized to either supervised Nordic walking, unsupervised Nordic walking or advice to remain active**.

For the pain scale of the LBPRS, all three groups showed small mean improvements during the intervention period (Figure 2). The mean improvement was 8.8 for the supervised NW group, 3.4 for the unsupervised NW group, and 4.8 for the advice to remain active group. These improvements were sustained at the 26 weeks time point for all three groups and at 52 weeks for the supervised NW group whereas a return towards baseline values was observed at 52 weeks for the unsupervised NW and the advice to remain active group. In the supervised NW group the improvement was significant at all time points (p = 0.009/0.01/0.03), in the unsupervised group the improvement was significant at 26 weeks and borderline at 11 and 52 weeks (p = 0.08/0.04/0.09), and in the advice to remain active group the improvement was significant except after 52 weeks (p = 0.04/0.01/0.18). However, no statistically significant differences between the groups were achieved at any of the time points even as indicated by the overlapping 95% confidence intervals though the supervised NW group consistently reported the largest average improvement (Figure 2).

**Figure 3:**
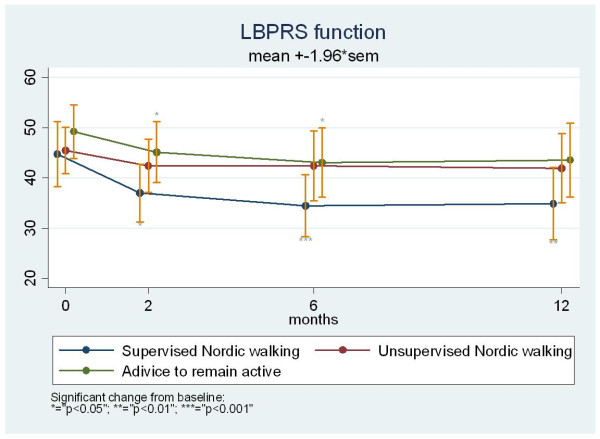
**Change over 12 months on the Low Back Pain Rating Scale (LBPRS) function subscale for chronic low back pain patients randomized to either supervised Nordic walking, unsupervised Nordic walking or advice to remain active**.

The same pattern was seen for the function scale of the LBPRS where the adjusted mean improvements at the eight week follow-up was 7.4 for the supervised NW group, 3.2 for the unsupervised NW group, and 3.8 for the advice to remain active group. In the supervised NW group the improvement was significant at all time points (p = 0.01/0.001/0.002), in the unsupervised NW group the improvement was borderline significant at 52 weeks and non-significant at 11 and 26 weeks (p = 0.14/0.20/0.06), and in the advice group the improvement was significant after 11 and 26 weeks and borderline significant after 52 weeks (p = 0.01/0.03/0.07). The supervised NW group continued to improve throughout the follow-up period, but still without any statistically significant differences between the groups as indicated by the overlapping 95% confidence intervals (Figure 3).

**Figure 4:**
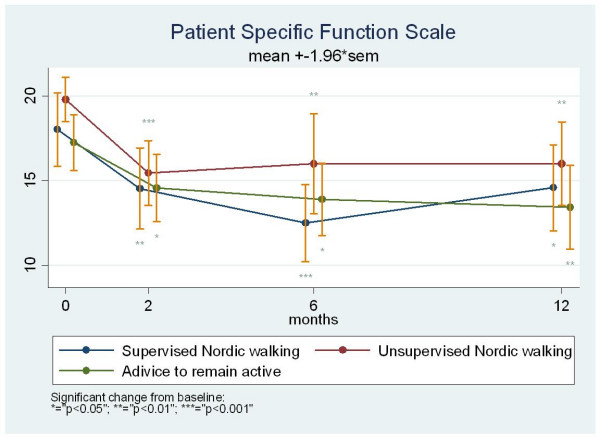
**Change over 12 months on the Patient Specific Function Scale for chronic low back pain patients randomized to either supervised Nordic walking, unsupervised Nordic walking or advice to remain active**.

For the PSFS, again no statistically significant differences between the groups were observed (Figure 4). However, within each group the improvement was significant at all time points post treatment (supervised NW group: p = 0.009/0.001/0.02, unsupervised NW group: p < 0.001/0.004/0.001, and advice to remain active group: p = 0.01/0.01/0.004).

The number of patients achieving the MCID on the pain subscale of the LBPRS was 10 in the supervised NW group, 10 in the unsupervised NW group, and 8 in the advice to remain active group. For the disability subscale of the LBPRS the corresponding proportions were 7, 4, and 4 one year after initiation of the intervention. Again this was not statistically significant between the groups.

### Secondary outcome measures

For the EQ5D, very small and practically identical adjusted mean changes were observed at all time points (data not shown).

The proportion of participants who changed from being on sick leave at baseline to not being in sick leave after the eight week intervention was 40% (six individuals) in the supervised NW group, 57% (12 individuals) in the unsupervised NW group, and 27% (seven individuals) in the advice to remain active group, but without statistical significance between the groups. Also use of over the counter pain medication or use of concurrent treatment during the one-year follow-up period were not statistically significantly different between the groups (data not shown).

Finally, patient expectations to treatment were significantly associated with improvement for all primary outcome measures.

### Accelerometers

**Figure 5:**
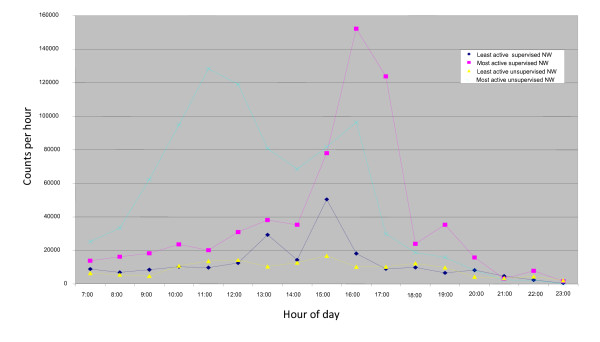
**Range of average intensity measured in counts per minute using Actigraph GT 256 accelerometers over a day for the most and least active individuals in the supervised and unsupervised Nordic walking groups**.

Altogether 25 (50.4%) of participants in the supervised NW group and 29 (65,2%) in the unsupervised NW group contributed data for seven or more days, the mean being 11 days out of the 14 in both groups. The average activity intensity over the entire day for the two groups was low and very similar for the period of measurement (5,000-5,5000 counts per hour corresponding to resting). In comparison active NW performed by a trained instructor was determined to result in an activity level of 240,000-300,000 counts per hour. There was however a wide range of activity levels in both groups as illustrated in Figure 5 but these could not be related to the back pain outcomes.

## Discussion

This randomized clinical trial demonstrates that eight weeks of bi-weekly supervised NW had small clinical effects and was no more effective than unsupervised NW or advice to remain active in chronic low back pain patients. However, the supervised NW group consistently reported greater averaged improvements for all primary outcome measures and a greater proportion of patients in this group achieved a clinically important improvement and none of the participants in the supervised NW group complained of negative side effects associated with the intervention. We further found that supervised NW twice a week for eight weeks resulted in no more average physical activity than unsupervised NW and that both groups had very low average daily activity levels.

Henchoz and So concluded in their recent systematic review that *exercise diminishes disability and pain severity while improving fitness and occupational status in patients who have subacute, recurrent, or chronic low back pain *[17]. Furthermore it has consistently been shown that exercise under supervision is more effective than non-supervised exercise [17,19,20]. Unfortunately however it is not known whether back specific exercises or general exercise in the form of for example NW is preferable in patients with chronic LBP since conflicting results are reported from primary studies [17,19]. For example Koumantakis et al found that general exercise alone reduced disability more than a combination of general exercise and trunk specific exercises [31] whereas Ferreira et al found that retraining of specific trunk muscles resulted in greater improvement in short term function than a general exercise program [32]. In all likelihood, specific sub-groups of patients respond differently to specific characteristics of exercise programs such as duration, intensity and frequency. We found that the average daily physical activity levels did not differ between the supervised NW group and the unsupervised NW group and this may explain the lack of statistically significant differences between the two groups.

A lower number of participants in the supervised group returned to work during the intervention compared to both of the other groups. One can speculate that this may be because these patients were busy participating in the trial and therefore not actively seeking employment contrary to patients in the other groups who were left without supervision. Of course the right time to measure whether participants were working or not would have been at the one-year follow-up. However, we only asked about sick listing immediately after the intervention period and that is indeed unfortunate.

Considering that the activity levels were so similar between the groups, the overall better average results in the supervised NW group were unlikely to be result of a physiological change. Rather we believe it was a result of fulfilled expectations and attention bias. We did find a statistically significant association between improvement in all three groups and participants' a priori expectations and generally patients had higher expectations to the supervised NW intervention.

This is the first study evaluation the effectiveness of NW in back pain patients and we have therefore learned important lessons in relation to this intervention. First and foremost, it was not easy to recruit participants and consequently the inclusion period was extended several times. Since NW is a novel and experimental treatment for back pain, patients may not have considered it to be a "real" or "serious" treatment for their chronic problem and highly motivated recruitment personnel is essential if future trials involving this intervention are undertaken. Second, there may have been a certain stigmatism around NW which in the public is often associated with elderly frail persons. Our target population was in their middle to late forties and may therefore have been reluctant to engage in such an activity. Interestingly and quite contrary to popular beliefs in Denmark, NW is also performed by young, highly trained and skilled individuals who compete at an international level and thus this intervention may be perceived differently in other countries [1]. Third, it is obvious based on the accelerometer data that the trained instructors were not able to motivate the participants in the supervised NW group to elevate their general physical activity level. In fact, some of the participants were not even able to comply with the very reasonable predetermined intensity during the NW sessions. Of course this lack of standardization is a weakness in this study but on the other hand may reflect a realistic picture of how NW would be performed under real-life circumstances outside the research setting. Researchers performing future studies evaluating the effect of this or similar interventions should therefore carefully consider how patient's motivation is dealt with. Fourthly, our inclusion criteria were quite broad and may have resulted in a heterogeneous group of participants. Finally, patients are referred to the back center from a rather large geographical area and travelling to the center twice a week for eight weeks was simply too inconvenient for many potential participants. In spite of these obstacles we did manage to recruit sufficient participants into the trial and to keep attrition to a minimum.

Statistical significant differences between groups may be a reflection of both the characteristics of the study sample and the sample size. Most patients referred to the back center have a long history of pain and disability and all have undergone a four-week period of treatment in the primary sector by either their family physician and/or a chiropractor and/or a physical therapist. Furthermore to be included into this trial they had to complete the standard four week treatment and exercise program at center and still have pain greater than three on the 11-box numeric rating scale (see inclusion criteria). Consequently they constitute a particularly motivation and treatment resistant sub-group and probably any improvement is worth the effort, in particular when the intervention is one with several other documented health benefits such as NW. Provided that a future sample is comparable to the present, 442 participants in each group are required to obtain a statistically significant difference on the LBPRS disability subscale between the supervised NW and the advice to remain active groups. Such a sample would be unusually high in back pain research but is not unheard of in other medical fields.

All groups experienced some mean improvement in pain and disability during the intervention period. The improvement in the advice to remain active group could possibly be a delayed effect of the four weeks of treatment and training at the back center and would thus account for some of the observed improvement also in the supervised NW group. The fact that the observed improvement in the pain and disability scores were maintained at the one-year follow-up (Figure 1 and 2) for the supervised NW group but not for the other groups is however likely a specific effect of the intervention or the attention/motivation from the instructors. Whether the eight weeks of supervised NW was enough to inspire a change of habit and whether this group continued to perform NW or whether the sustained improvement is due to a long term physiological effect is unknown.

## Conclusions

We found no statistically significant effect in chronic LBP patients of supervised NW when this was compared to unsupervised NW or advice to remain active in a randomized clinical trial. It was not possible to motivate participants in the supervised NW group to become more physically active on the average. The mean improvements were, however, generally greater for the supervised NW group compared to the other two groups. The explanations are likely a larger expectation to the treatment and more contact with health care personnel. It is also possible that a subgroup of patients actually did benefit and that NW may prove to be a safe and cheap intervention with some effect in selected groups of LBP patients.

## Competing interests

The authors declare that they have no competing interests.

## Authors' contributions

All authors participated in the design of the study. JH secured the funding and was the primary investigator. LM was project manager. JH, TB and CM participated in the analysis and interpretation of findings. JH wrote the manuscript draft. All authors commented on and approved the final version.

## Pre-publication history

The pre-publication history for this paper can be accessed here:

http://www.biomedcentral.com/1471-2474/11/30/prepub
